# Retraction ‘Reduced interleukin-38 in non-small cell lung cancer is associated with tumour progression’

**DOI:** 10.1098/rsob.200204

**Published:** 2020-07-22

**Authors:** Feng Wang, Weihua Zhang, Tianfeng Wu, Heying Chu

*Open Biol.* **8**, 180132. (Published 31 October 2018). (doi:10.1098/rsob.180132)

The *Editor-in-Chief* in agreement with the Publisher has retracted this article. Following an investigation, we have found that the published manuscript has substantial overlap with a previously published manuscript [1] in the text and figures (notably figure 1 Kaplan–Meier curves, table values, line graphs and figure 3 western blots). This paper has been identified among of a set of papers that present significant text and data duplication and therefore the data reported in this article are unreliable. The authors are in agreement with this retraction.

Corresponding authors Yu Zhouxu (YuZhouxu@aliyun.com) Feng Wang (FengWang@163.com)

Prof. Jon Pines FRS *Editor-in-Chief, Open Biology*
